# Semimetallic, Half-Metallic, Semiconducting, and Metallic States in Gd-Sb Compounds

**DOI:** 10.3390/ijms24108778

**Published:** 2023-05-15

**Authors:** Semyon T. Baidak, Alexey V. Lukoyanov

**Affiliations:** 1 Institute of Physics and Technology, Ural Federal University Named after the First President of Russia B.N. Yeltsin, 620002 Ekaterinburg, Russia; baidak@imp.uran.ru; 2 M.N. Mikheev Institute of Metal Physics of Ural Branch of Russian Academy of Sciences, 620108 Ekaterinburg, Russia

**Keywords:** electronic structure, topologic structure, alloys, intermetallic compounds, first principles calculations

## Abstract

The electronic and band structures of the Gd- and Sb-based intermetallic materials have been explored using the theoretical ab initio approach, accounting for strong electron correlations of the Gd-4f electrons. Some of these compounds are being actively investigated because of topological features in these quantum materials. Five compounds were investigated theoretically in this work to demonstrate the variety of electronic properties in the Gd-Sb-based family: GdSb, GdNiSb, Gd_4_Sb_3_, GdSbS_2_O, and GdSb_2_. The GdSb compound is a semimetal with the topological nonsymmetric electron pocket along the high-symmetry points Γ–X–W, and hole pockets along the L–Γ–X path. Our calculations show that the addition of nickel to the system results in the energy gap, and we obtained a semiconductor with indirect gap of 0.38 eV for the GdNiSb intermetallic compound. However, a quite different electronic structure has been found in the chemical composition Gd_4_Sb_3_; this compound is a half-metal with the energy gap of 0.67 eV only in the minority spin projection. The molecular GdSbS_2_O compound with S and O in it is found to be a semiconductor with a small indirect gap. The GdSb_2_ intermetallic compound is found to have a metallic state in the electronic structure; remarkably, the band structure of GdSb_2_ has a Dirac-cone-like feature near the Fermi energy between high-symmetry points Г and S, and these two Dirac cones are split by spin-orbit coupling. Thus, studying the electronic and band structure of several reported and new Gd-Sb compounds revealed a variety of the semimetallic, half-metallic, semiconducting, or metallic states, as well topological features in some of them. The latter can lead to outstanding transport and magnetic properties, such as a large magnetoresistance, which makes Gd-Sb-based materials very promising for applications.

## 1. Introduction

Quantum materials [1] include wide classes of topological materials [2]. These materials resulted in tremendous research efforts and attracted interest because of their exotic physics [3,4] and the various exciting applications of insulators, superconductors, metals, or semimetals [5]. A number of those topological compounds contain antimony Sb [6]. Additionally, the Sb-containing compounds have exhibited superconducting [7] or thermoelectric properties [8].

Recently, a family of the binary RX compounds, where R designates a rare-earth metal and X is a s/p element, attracted newfound attention for their unusual electronic properties, with topological features in some such compounds. Research for electronic states with non-trivial topology in the RX compounds started in 2016 with the study of LaBi, where Dirac-cone-like features were found [9,10,11]. Although some studies reported the absence of band inversion and suggested the trivial topology of electronic states in some similar compounds, such as LaSb, CeSb, LuBi, and YBi [12,13]. In another work on the LaBi compound, the nontrivial band anticrossing along the Г–X direction and two distinct topological surface states were identified [14]. Then, angle-resolved photoemission spectroscopy (ARPES) measurements for a whole series of RSb compounds were performed and revealed two hole and two electron pockets at high-symmetry points Г and X, respectively [15]. Another study predicted topologically non-trivial states, such as band inversion and the presence of Weyl fermions in the band structure of GdSb and GdBi [16]. Both these compounds are ordered antiferromagnetically below 23.4 and 25.8 K, respectively [17]. Experimental optical studies of GdSb and TbSb revealed an anomalous behavior of the optical conductivity dependences in the low-energy region, the semimetallic type of the conductivity of these compounds, predicted by calculations of the density of states, is confirmed by optical studies [18]. The existence of electronic states with non-trivial topology in the band structure of compounds can lead to unusual magnetic properties, such as extremely large unsaturated magnetoresistance (XMR) [19], and some even report this being the case for the LuSb compound [20], which is why it is such a popular topic of interest. Large magnetoresistance has also been observed in other compounds from the rare-earth monoantimonide family, such as TbSb and HoSb [21,22,23].

By adding one more element, T, which stands for a transition metal, to the family of binary RX compounds, one obtains another family of ternary RTX compounds with a variety of different properties [24,25,26]. One can observe a Dirac-like state at the high-symmetry point X, which is very close to the RTX family compound GdSbTe [27,28]. Electronic structure calculations for some compounds from the family were conducted, and they show that the low-temperature phase of the GdNiSb compound is a narrow-gap semiconductor [29,30] and GdPtSb is metallic in nature [30]. Some of the other RTX compounds, such as a half-Heusler GdPtBi alloy, similarly to the binary RX compounds, show the semimetallic behavior and host Weyl points in the magnetic field [31,32]. In this work, RNiSb compounds were investigated in terms of magnetic properties, and it was found that most of them show Curie–Weiss behavior with magnetic ordering temperatures below 4 K [33], and magnetism is dominated by the magnetic moments of rare-earth elements [34]. These ternary RNiSb compounds also have remarkable thermoelectric properties [35,36,37], with the highest thermoelectric figure of merit ZT being up to ~0.7 for a wide range of temperatures in ErNiSb [37]. The electronic structure of ErNiSb was reported in [38] and the indirect band gap 0.25 eV was confirmed.

While the equiatomic Gd-Sb compounds have already been investigated using various experimental and theoretical methods, the non-equiatomic Gd-Sb compounds have been studied quite poorly. Some of the rare-earth diantimonides were synthesized with a LaSb_2_ type of structure and high-pressure orthorhombic type of structure, see [39] and references. The Gd-Sb system was also studied in another work using high-temperature differential thermal analysis (DTA) [40]; four compounds were formed, GdSb, GdSb_2_, Gd_4_Sb_3,_ and Gd_5_Sb_3_. Another interesting Gd-Sb compound, namely, GdSbS_2_O, belongs to the LnSbS_2_O family; it was reported as having been synthesized with the p-type semiconductor properties [41]. It is similar in its structure and composition to the series of molecular Gd_2_SO_2_-based doped compounds, which found numerous applications in cold neutron imaging as ultrathin screens [42] and in the biomedical field [43] because of their controllable particle size [44] and photoluminescence properties [43].

In this work, we investigate and compare the electronic structure and magnetic properties of several intermetallic Gd-Sb compounds and discuss topological features in the band structure. The revealed variety of the semimetallic, half-metallic, semiconducting, or metallic states can be related with different anomalous properties of these compounds. 

## 2. Results and Discussion

### 2.1. GdSb Compound

**Figure 1:**
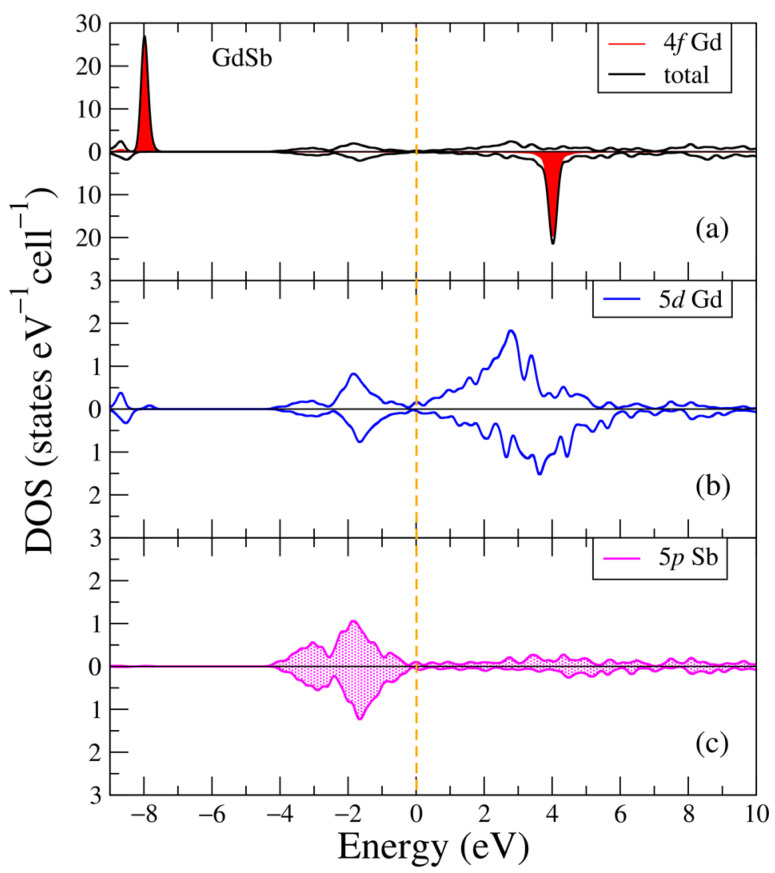
Electronic structure of GdSb: (**a**) Total and partial Gd-4f densities of states; (**b**) Partial density of states for Gd-5d; (**c**) Partial density of states for Sb-5p from states from density functional theory (DFT)+U [18]. The plot is shifted relative to the Fermi energy, shown at zero as a vertical line.

In Figure 1, the electronic structure of the GdSb intermetallic compound is plotted for two opposite spin directions. Two intense peaks in the total density of states (DOS) for the majority and minority spin directions of GdSb in Figure 1a are composed of the 4f Gd states at the following energies: about −8 eV and 4 eV below the valence and inside the conduction bands, respectively. These Gd-4f states result in the moment of the Gd ion being 7.16 $\mu_{B}$, in GdSb the magnetic moments of the Gd ions compensate each other and form an antiferromagnetic ordering with almost a zero value of magnetization [45]. The magnetic moment of the GdSb binary compound is composed of the gadolinium ion moment, whereas the stibium ion in this compound is calculated to be negligible. The close value of the total moment for Gd in GdSb is equal to 6.98 $\mu_{B}$, and it was calculated for the most relevant set of the U and J parameters in the previous local density approximation (LDA)+U calculations [46]. Additionally, in [46], similar positions (about −8 eV and 4 eV) of the occupied 4f bands are found for fitting with spectra and HSE06 hybrid functional calculations [47].

One can note the contributions of Gd-5d and Sb-5p states in the valence band of the GdSb compound, as seen in Figure 1b,c. For both spin projections, the Gd-5d electronic states are located in the conduction band, and are mostly not occupied (Figure 1b). In [18], it was discussed that these energy positions of the electronic states allow one to reproduce and interpret the semimetallic behavior of the experimental optical conductivity curve for GdSb. The other electronic states (not shown in Figure 1) have negligible contributions.

**Figure 2:**
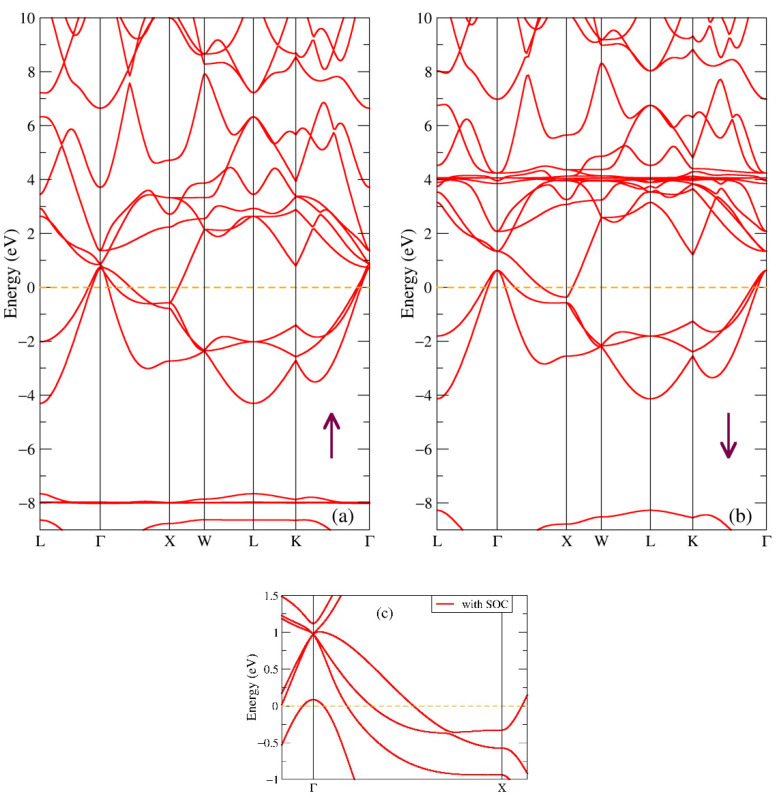
Band structure of GdSb: (**a**) Majority (up arrow); (**b**) Minority (down arrow) spin projections from DFT+U; (**c**) With spin-orbit coupling [18].

**Figure 3:**
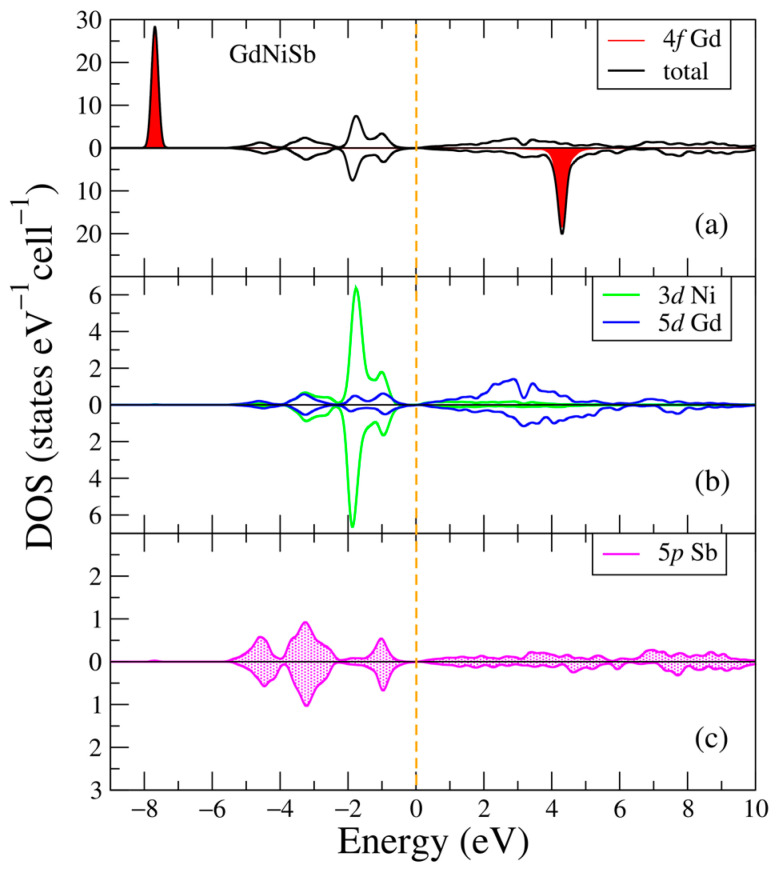
Densities of electronic states from DFT+U for GdNiSb. (**a**) Total and partial Gd-4f densities of states; (**b**) Partial density of states for Gd-5d, Ni-3d; (**c**) Partial density of states for Sb-5p. The plot is shifted relative to the Fermi energy, shown at zero as a vertical dashed line.

In Figure 2, one can see the band structure of both spin projections of the binary GdSb compound. It is obvious in the figure that this phase is gapless with the valence and conduction bands nearly touching. Bands near the Г point from the valence band spread above the Fermi level creating a hole pocket, same with bands from the conduction band, which create an electron pocket near the X point; such features are typical for semimetals [18]. There are noticeable flat bands at energies −8.0 eV for one spin direction, see Figure 2a, and 4 eV for other spin direction, see Figure 2b, which correspond to the Gd-4f intense peaks with the same energies plotted in red in Figure 1a. One can also notice few points with degenerate bands near the Fermi energy, moving from the Г high-symmetry point to X for the majority spin projection, as it is clearly seen in Figure 2a. The similar semimetallic band structure was obtained using HSE06 hybrid functional without spin-orbit coupling [47]. The Figure 2c panel shows a small part of the band structure near the Fermi level with spin-orbit coupling (SOC). The degeneration at point X is lifted, and one of the bands at point Г shifted a few tenths of eV lower. In the HSE06 hybrid, functional with SOC [47], the changes of the bands are very similar; there the difference in the degeneracy of the bands at the Г point and the electronic pocket at X are closer to the Fermi energy. The very recent ARPES measurements for a 20 nm thick GdSb magnetic semimetal film [48] revealed an ellipsoidal electron pocket at the bulk X point, two nearly spherical light-hole spin-orbit split-off bands at the bulk Г point, and a warped heavy-hole band resembling a square Fermi surface. The authors of [48] used the DFT+U band structure, which is very similar to the one depicted in Figure 2c, to interpret the ARPES bands; however, the band dispersion in Figure 2c with SOC can better reproduce the β and δ bands (Figure 3 and Figure 4 in [48]) with the valence and conduction bands almost touching near point X.

### 2.2. GdNiSb Compound

In Figure 3, the electronic structure of the GdNiSb intermetallic compound is plotted for two opposite spin directions. Similar to Figure 1a, one can see two intense peaks from the 4f Gd states being a little bit higher than in the binary GdSb compound energies: −7.7 eV and 4.3 eV. Two other noticeable intense peaks lay at −1.9 eV for both spin projections, and belong to the electronic states of the occupied 3d Ni shell, similar to the position of this peak in the calculations [35]. The valence band here mostly comprises the 3d Ni states with the smaller contributions from the 5d Gd and 5p Sb states, and the main contributors to the conduction band are the 5d Gd states, see Figure 3b,c. All shown electronic states except 4f Gd are non-polarized. The other electronic states (not shown in Figure 3) have negligible contributions. This picture of the electronic states is different from local spin-density approximation (LSDA) [29] because the corrections for strong electron correlations and SOC are required for the 4f shell and bands near the Fermi energy. The calculations resulted in the magnetic moment being 7.20 $\mu_{B}$ in GdNiSb, the magnetization measured being 8.1(5) $\mu_{B}$ [33], and previous theoretical calculations being 7.00 [29,30] and 6.949 $\mu_{B}$ [35]. 

**Figure 4:**
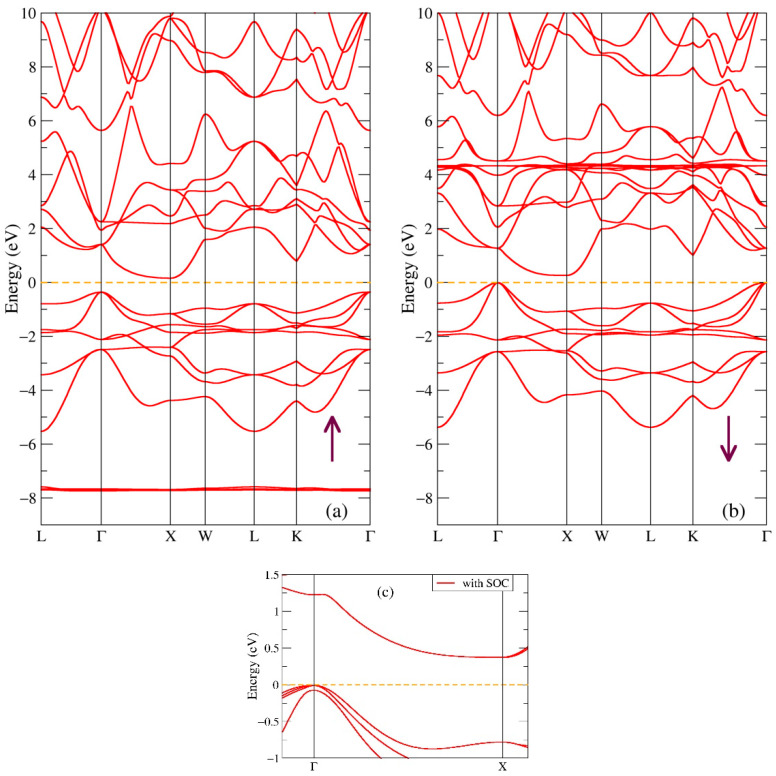
Band structure of GdNiSb: (**a**) Majority (up arrow); (**b**) Minority (down arrow) spin projections. Panel (**c**) shows the effect of spin-orbit coupling in GdNiSb.

In Figure 4 one can see the band structure of both spin projections of the ternary GdNiSb compound. The first observation is that the addition of nickel to the GdSb formula opens the gap in the band structure with the value of 0.26 eV in the minority spin direction, as calculated from the band structure, see Figure 4b. Indirect band gaps of 0.292 [30] and 0.279 eV [35] in GdNiSb were also found earlier in the similar band structure obtained in the FP-LAPW(+lo, version 19) Wien2k calculations. At the same time, the distance between the occupied and empty states is equal to 0.52 eV for the majority spin direction, Figure 4a. The high-symmetry point Г is still the point with the highest energy from the valence band, and there still could be some kind of a hole pocket but much smaller than in the GdSb compound. The same feature can be found at the high-symmetry point X with the lowest energy from the conduction band. Such band structure features indicate that the ternary GdNiSb compound is a semiconductor with an indirect gap of 0.26 eV, as calculated from the band structure. Similar to Figure 2, there are flat bands at energies −7.7 eV for one spin direction in Figure 4a and at 4.3 eV for the other spin direction (Figure 4b), which correspond to the Gd-4f intense peaks, see Figure 3a. Panel c shows a small part of the band structure near the Fermi level with spin-orbit coupling taken into account. Such an effect mostly manifests itself by a slight increase of the energy gap value from 0.26 to 0.38 eV. The experimental value of the band gap in GdNiSb has not been reported in the literature; this value is close to the one in RNiSb [34].

### 2.3. Gd_4_Sb_3_ Compound

**Figure 5:**
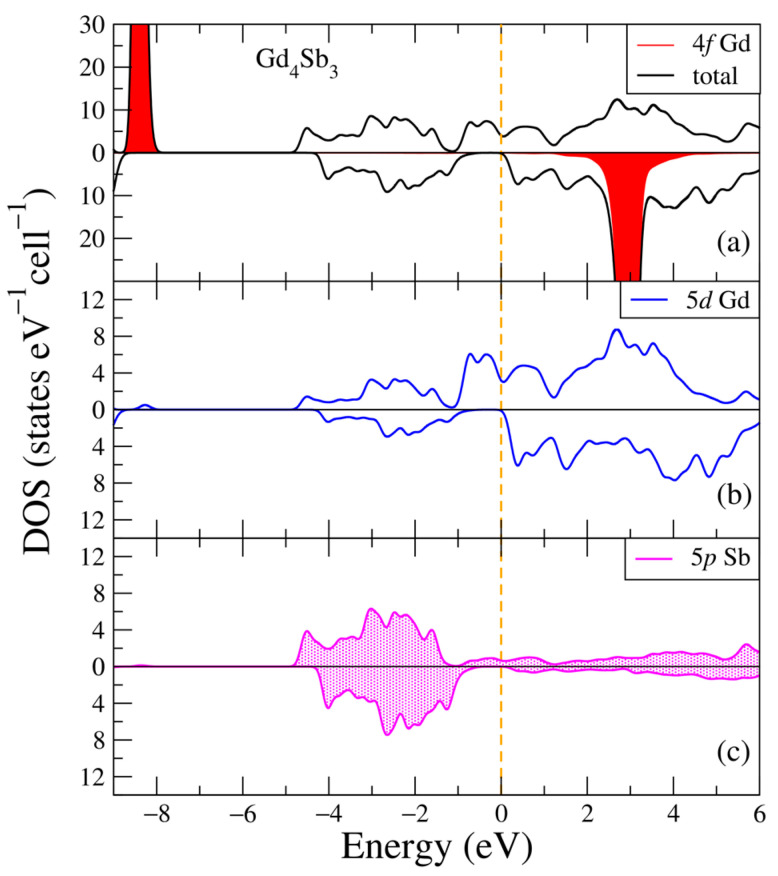
Densities of electronic states from DFT+U for Gd_4_Sb_3_. (**a**) Total and partial Gd-4f densities of states; (**b**) Partial density of states for Gd-5d; (**c**) Partial density of states for Sb-5p. The plot is shifted relative to the Fermi energy, shown at zero as a vertical line.

In Figure 5, the electronic structure of the Gd_4_Sb_3_ intermetallic compound is plotted for two opposite spin directions. Similar to Figure 1a and Figure 3a, one can see two intense peaks from the 4f Gd states at the following energies: −8.4 eV and 3.0 eV. The valence band is composed of the 5p Sb states and less amount of the 5d Gd states, and the main contributors to the conduction band are the 5d Gd states, see Figure 5b,c. One can notice that the density of states is quite high around the Fermi energy for the majority spin projection, Figure 5a,b which suggests the metallic nature of the compound, but in the minority spin projection, there is an obvious energy gap. Such a band structure is typical of half-metals, and it is a key feature of this Gd-bearing compound. The other electronic states (not shown in Figure 5) have negligible contributions.

**Figure 6:**
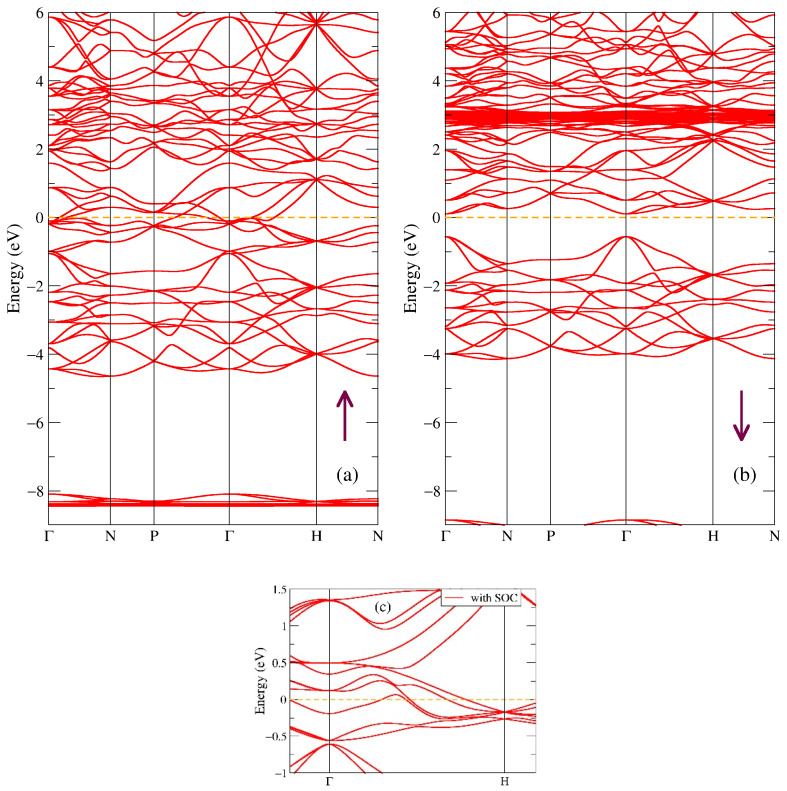
Band structure of Gd_4_Sb_3_: (**a**) majority (up arrow) and (**b**) minority (down arrow) spin projections. Panel (**c**) shows the effect of spin-orbit coupling in Gd_4_Sb_3_.

In Figure 6, one can see the band structure of both spin projections of the Gd_4_Sb_3_ compound. The change of stoichiometry in Gd-Sb from 1:1 (GdSb) to 4:3 (Gd_4_Sb_3_) results in opening the energy gap in the minority spin projection (Figure 6b), and vice versa, increasing the density of states near the Fermi energy in the majority spin projection, Figure 6a. Such a band structure is a good indicator that the compound in question is a half-metal. The energy gap in the minority spin direction, see Figure 6b, is a direct gap with the smallest value of 0.67 eV at the Г high-symmetry point, as it is estimated from the band structure. The band structure of the majority spin direction is metallic, with a number of crossing bands near the Fermi level. Similar to Figure 2 and Figure 4, there are localized states at energies corresponding to the Gd-4f intense peaks, as can be seen in Figure 5a. Panel c in Figure 6 shows a small part of the band structure near the Fermi level with the spin-orbit coupling taken into account in a separate calculation. The main effect of SOC is seen in this panel as a lift of the degeneration at the Г point right below the Fermi energy and a shift of the batch of bands at H point (by a few tenths of eV higher) closer to the Fermi level. The total magnetic moment of Gd_4_Sb_3_ is obtained in our calculations as 31.00 $\mu_{B}$/f.u.; no experimental value has been reported in the literature for this compound. In the Gd_5_Sb_3_ compound, no half-metallic state has been found; the metallic DOS is calculated for both spin projections [49].

### 2.4. GdSbS_2_O Compound

**Figure 7:**
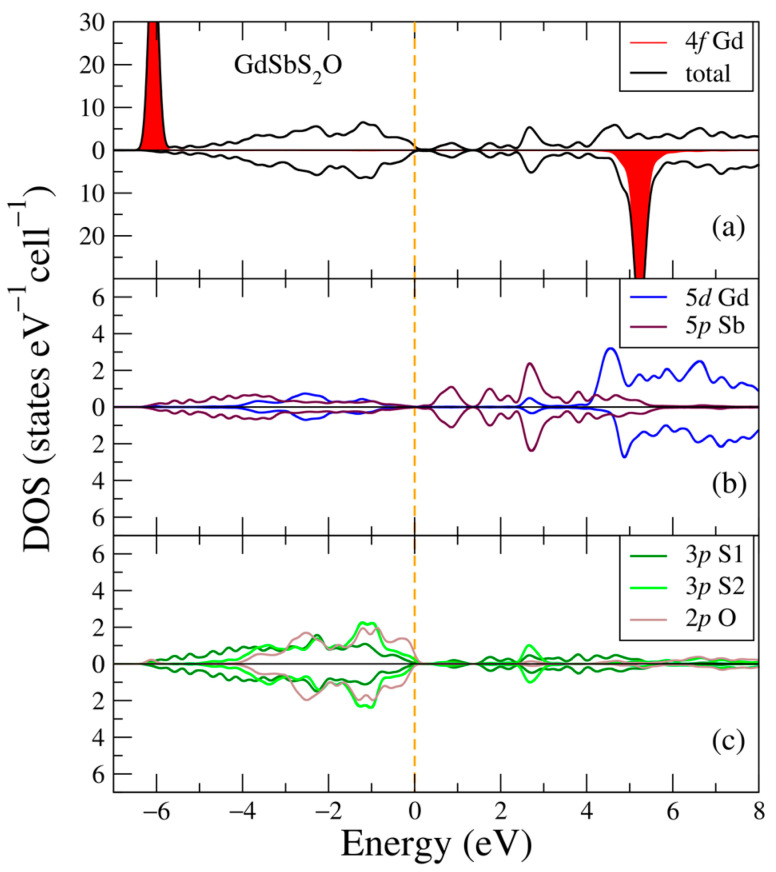
Densities of electronic states from DFT+U for GdSbS_2_O. (**a**) Total and partial Gd-4f densities of states; (**b**) Partial density of states for Gd-5d, Sb-5p; (**c**) Partial density of states for S1-3p, S2-3p, O-2p. The plot is shifted relative to the Fermi energy, shown at zero as a vertical line.

In Figure 7, the electronic structure of the GdSbS_2_O compound is plotted for two opposite spin directions. Similar to Figure 1a, Figure 3a and Figure 5a, one can see two intense peaks from the 4f Gd states at energies −6.1 and 5.2 eV, which are higher than in previous compounds. The main contributors to the valence band here are the 3p S1, S2, and 2p O electronic states added to the structure with S and O atoms. The conduction band is now mostly made of the 5p Sb electronic states, which built the valence band in the three previous compounds, and the 5d Gd electronic states moved to higher energies. The other electronic states (not shown in Figure 7) have negligible contributions. One can notice what looks like the absence of electronic states between valence and conduction bands, which suggests that there might be a small gap in the band structure. 

**Figure 8:**
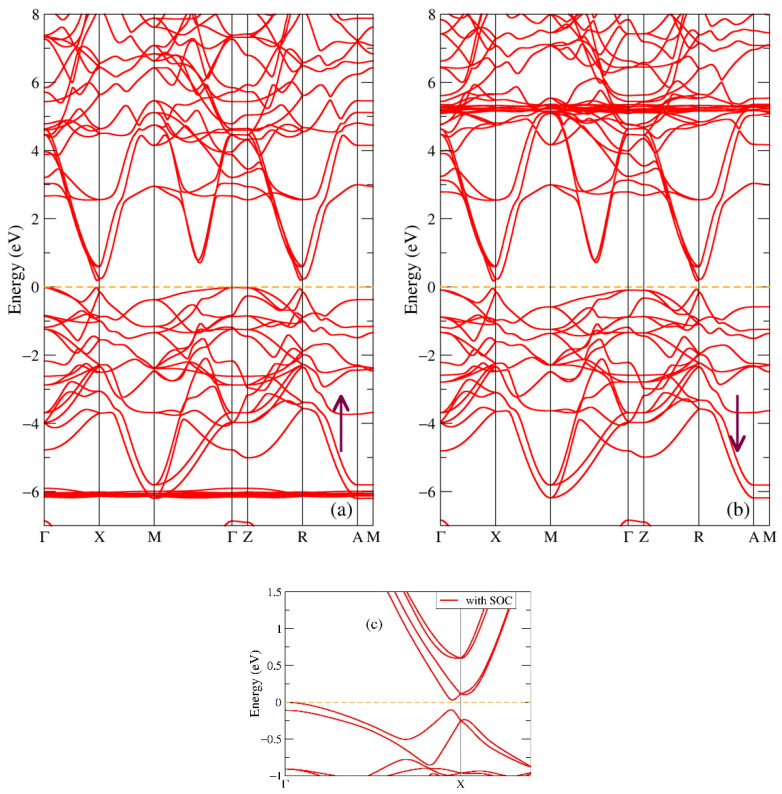
Band structure of GdSbS_2_O: (**a**) majority (up arrow) and (**b**) minority (down arrow) spin projections. Panel (**c**) shows the effect of spin-orbit coupling.

In Figure 8, one can see the band structure of both spin projections of the GdSbS_2_O compound. As we guessed from the densities of the states, the addition of S_2_O to the GdSb formula opens a gap with the value 0.20 eV in the band structure in the majority spin direction, see Figure 8a. At the same time, the distance between the occupied and empty states is equal to 0.21 eV for the minority spin direction, Figure 8b. The smallest value of the energy gap is between two points close to the high-symmetry point X (not exactly at the X point), which suggests that GdSbS_2_O is a semiconductor with a direct gap of 0.20 eV, as it is estimated from the band structure. From the electrical conductivity of GdSbS_2_O, a thermal gap of 1 eV [41] was reported; there are no other experimental data for this molecular compound. Similar to Figure 2, Figure 4 and Figure 6, there are flat bands at energies −6.1 eV for one spin direction in Figure 8a, and at 5.2 eV for the other spin direction (Figure 8b), which correspond to the Gd-4f intense peaks, see Figure 7a. Figure 7c shows a small part of the band structure near the Fermi level when spin-orbit coupling is taken into account in additional calculations. The effect of SOC in this situation significantly reduces the gap value from 0.20 to 0.03 eV and changes the direct gap to an indirect gap, with the new point of highest energy from the valence band at the high-symmetry point Г, which indicates that GdSbS_2_O is actually a semiconductor with the small indirect gap of 0.03 eV. 

Additional calculations were performed for this molecular compound, and a Van der Waals correction was included because of the layered crystal structure of the GdSbS_2_O compound. The impact of long-range interactions is found to be negligible with the change of the energy gap in the order of ~10^−3^ eV. The total magnetic moment of GdSbS_2_O is obtained in our calculations as 6.98 $\mu_{B}$/f.u.; no experimental value has been reported in the literature.

### 2.5. GdSb_2_ Compound

**Figure 9:**
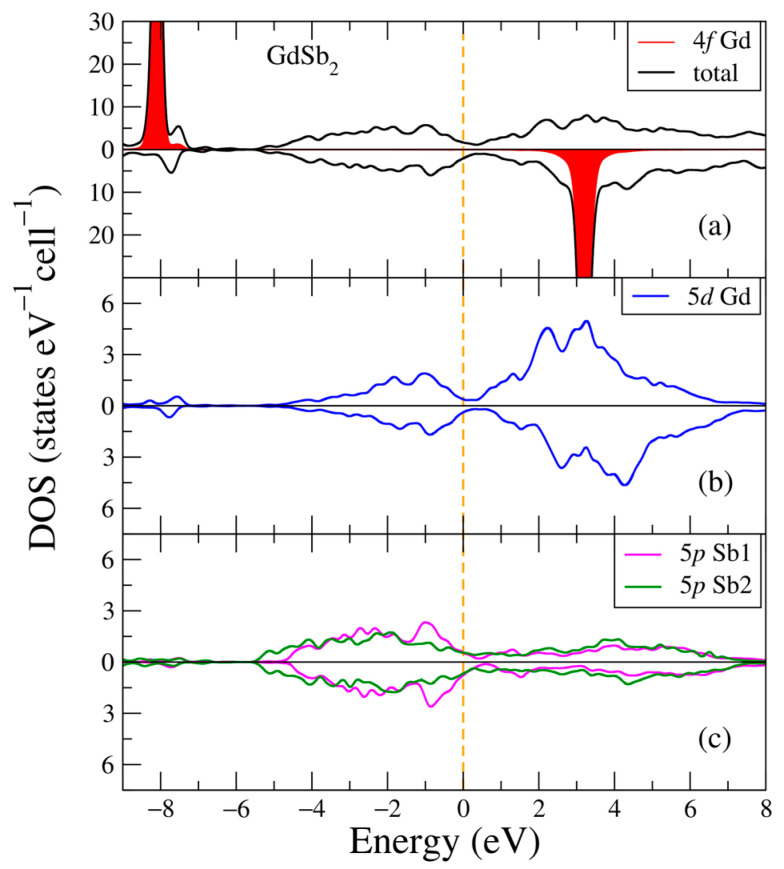
Densities of electronic states from DFT+U for GdSb_2_. (**a**) Total and partial Gd-4f densities of states; (**b**) Partial density of states for Gd-5d; (**c**) Partial density of states for Sb1-5p, Sb2-5p. The plot is shifted relatively to the Fermi energy, shown at zero as a vertical line.

In Figure 9, the electronic structure of the GdSb_2_ intermetallic compound is plotted for the two opposite spin directions. Similar to the previous electronic structures, one can see two intense peaks from the 4f Gd states at the following energies: −8.1 eV and 3.2 eV. The valence band is composed of the hybridized 5p Sb1, Sb2, and 5d Gd electronic states, and the main contributors to the conduction band are the 5d Gd electronic states, see Figure 9b,c. One can notice that there is non-zero density of states around the Fermi energy with the valence and conduction bands overlapping for both spin projections Figure 9a suggests the metallic nature of the compound. The other electronic states (not shown in Figure 9) have negligible contributions.

**Figure 10:**
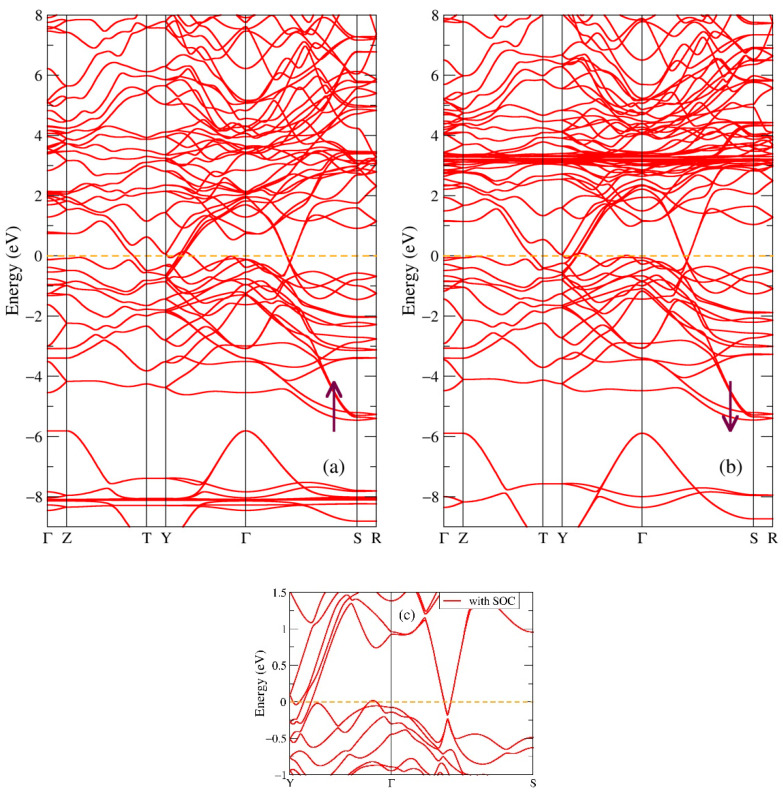
Band structure of GdSb_2_: (**a**) majority (up arrow) and (**b**) minority (down arrow) spin projections. Panel (**c**) shows the effect of spin-orbit coupling in GdSb_2_.

In Figure 10, one can see the band structure of both spin projections of the GdSb_2_ compound. The change of stoichiometry in Gd-Sb from 1:1 (GdSb) to 1:2 (GdSb_2_) results in the overlapping of the valence and conduction bands with several crossings of the Fermi level around T and Y high-symmetry points. However, the other parts of the band structure still maintain some distance between occupied and empty electronic states, which leads to the small but non-zero density of states around the Fermi energy, see Figure 9a. These features of the band and electronic structures indicate that this compound is a metal. Like in all above investigated compounds, there are localized states at energies corresponding to the Gd-4f intense peaks, as can be seen in Figure 9a. One can also notice a Dirac-cone-like feature near the Fermi level between high-symmetry points Г and S. Panel c in Figure 10 shows a small part of the band structure near the Fermi level with spin-orbit coupling taken into account in a separate calculation. The effect of SOC brings no significant changes in the band structure, although there is a small splitting of the two Dirac cones in the Г–S high-symmetry direction. The total magnetic moment of GdSb_2_ is obtained in our calculations as 7.03 $\mu_{B}$/f.u.; to our knowledge, no experimental value has been reported in the literature. In binary rare-earth pnictide compounds, topological features are related with outstanding spectral and magnetic properties, including large magnetoresistance [19,20]; thus, additional experimental studies on GdSb_2_ are required.

## 3. Materials and Methods

**Figure 11:**
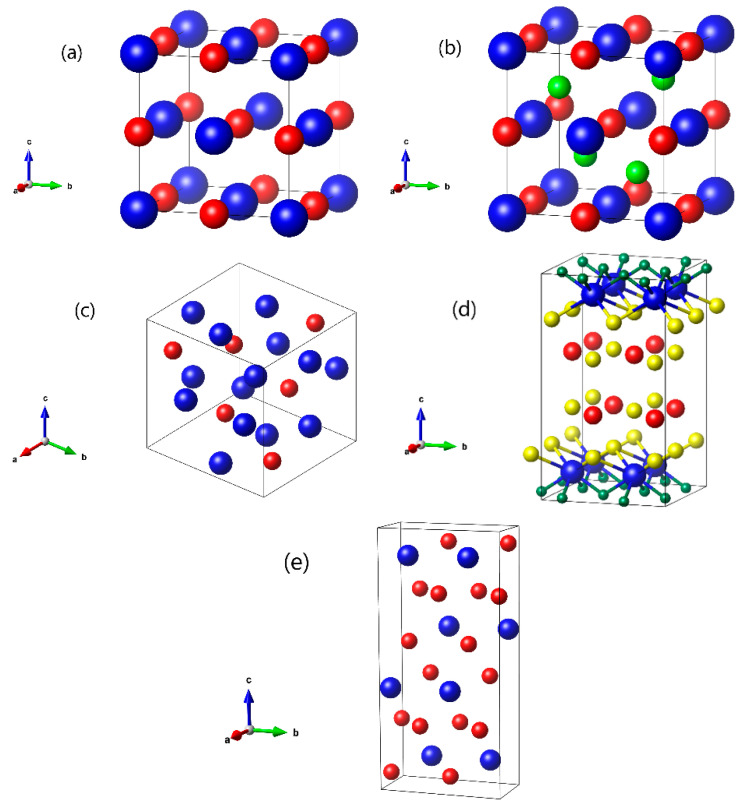
Crystal structure of: (**a**) GdSb; (**b**) GdNiSb; (**c**) Gd_4_Sb_3_; (**d**) GdSbS_2_O; (**e**) GdSb_2_ compounds plotted in Vesta [50]. Gd atoms are shown in blue, Ni in green, Sb in red, S in yellow, and O in dark green.

The crystal structure motives of all five compounds are plotted in Vesta [50] in Figure 11. Lattice parameters and atomic positions are shown in Table 1 below. One can see that the Sb atom in the ternary GdNiSb (b) has an environment of four Ni atoms in the form of a tetrahedron, and if one removes the Ni atoms from it, the exact binary GdSb (a) structure is obtained. In the molecular compound GdSbS_2_O (d), one can notice a layered structure with the alternation of two Sb–S and one S–Gd–O layers. The GdSb_2_ (e) compound also has a layered structure with the alternation of two distorted Gd–Sb layers and one Sb layer with square lattice, where one distorted Gd–Sb layer is actually two separate square Gd and Sb lattices very close to each other.

In this work, band and electronic structures were calculated using the Quantum Espresso program set [53,54], taking into account correlation effects as LSDA(GGA)+U [55]. This method is justified for strong electron correlations taken in the 4f shells of rare-earth elements. The exchange correlation functional form was used in the generalized gradient approximation (GGA) of J.P. Perdew, K. Burke, and M. Ernzerhof (PBE-version) [56]. 

In our GGA+U calculations, the exchange (Hund) parameter J as 0.7 eV and direct Coulomb interaction U as 6.7 eV were used for Gd as in previous works [55,57,58]. In this work we assume that the magnetic moments of rare-earth elements have ferromagnetic ordering. In our study, the standard potentials from the library of pseudopotential (Quantum ESPRESSO) for Ni, Sb, S, and O [59], as well as the projected augmented wave method (PAW) scalar relativistic potentials for gadolinium [60]. These pseudopotentials were used in our calculations, with the electronic configurations for generation given in parentheses [61]. One should note that all-electron full-potential codes, such as the Elk code [62], may be more precise in total energy and other parameters than pseudopotential-based codes. This is important for magnetic properties, e.g., for Gd_3_Cu_3_Sb_4_ in [63]. On the other hand, the systematic comparisons of different calculated values, including band gap, revealed that modern pseudopotentials can provide similar precision [64]. Wave functions were expanded in plane waves; we used a smearing of 0.01 Ry in the Marzari–Vanderbilt–De Vita–Payne smearing scheme [61] in Brillouin-zone integration for a 12 × 12 × 12 **k**-mesh. The method of plane-augmented waves was taken to include interactions between ions and valence electrons. SOC was additionally accounted for within full relativistic ultrasoft pseudopotentials from the standard Quantum Espresso library [59], listed here [61]. In additional calculations, long-range interactions (Van der Waals forces) were considered for the layered GdSbS_2_O compound with many-body dispersion (MBD) correction [65].

## 4. Conclusions

In this work, we explored different Gd-Sb materials for electronic and band structure features. The compounds based on these two elements are being actively investigated because of topological features and the large magnetic moment. Five compounds were investigated theoretically to demonstrate the variety of electronic properties in these Gd-Sb-based compounds, namely, GdSb, GdNiSb, Gd_4_Sb_3_, GdSbS_2_O, and GdSb_2_. We started with the main representative from the Gd-Sb system, which is the GdSb compound with the semimetallic properties and topological nonsymmetric electron pocket along Γ–X–W and the hole pockets in the L–Γ–X path. Our calculations show that the addition of nickel atoms to the system results in the energy gap, and we obtain a semiconductor with an indirect gap of 0.38 eV for the GdNiSb intermetallic compound. By changing chemical composition from 1:1 (GdSb) to 4:3 (Gd_4_Sb_3_), one can obtain a half-metal with the energy gap of 0.67 eV only in the minority spin projection. The GdSbS_2_O compound with S and O in its composition is found to be a semiconductor with the small indirect gap. GdSb_2_ is the last compound in this series, which has a metallic nature, with overlapping valence and conduction bands, as it has been shown with calculated electronic and band structures. Nevertheless, the band structure of GdSb_2_ has a Dirac-cone-like feature near the Fermi energy between high-symmetry points Г and S; these two Dirac cones are split by spin-orbit coupling. Additional experimental studies of the Gd-Sb-based compounds are required. Thus, the semimetallic, half-metallic, semiconducting, or metallic states in the electronic structure, as well as the topological features in the band structure and the large magnetic moment from the rare-earth element demonstrate that the combination of Gd and Sb in quantum materials may result in very promising properties for applications.

## Figures and Tables

**Table 1:** Lattice parameters and atomic positions for GdSb, GdNiSb, Gd_4_Sb_3_, GdSbS_2_O, and GdSb_2_ compounds.

Compound	Space Group (No.)	Lattice Parameters (Å)			Atomic Positions				
		a	b	c	Element	Wyckoff Symbol	X	Y	Z
GdSb [51]	225	6.210	6.210	6.210	Gd	4*a*	0	0	0
					Sb	4*b*	0.5	0.5	0.5
GdNiSb [33,52]	216	6.324	6.324	6.324	Gd	4*a*	0	0	0
					Ni	4*c*	0.25	0.25	0.25
					Sb	4*b*	0.5	0.5	0.5
Gd_4_Sb_3_ [40]	220	9.220	9.220	9.220	Gd	16*c*	0.0833	0.0833	0.0833
					Sb	12*a*	0.375	0	0.25
GdSbS_2_O [41]	129	3.900	3.900	13.700	Gd	2*c*	0.25	0.25	0.0936
					Sb	2*c*	0.25	0.25	0.6263
					S1	2*c*	0.25	0.25	0.379
					S2	2*c*	0.25	0.25	0.813
					O	2*a*	0.75	0.25	0
GdSb_2_ [39]	64	6.157	5.986	17.830	Gd	8*f*	0	0.1347	0.3902
					Sb1	8*f*	0	0.1314	0.0640
					Sb2	8*e*	0.25	0.3778	0.25

## Data Availability

The data presented in this study are available on request from the corresponding author.
